# Editorial: globalisation and the journal of eating disorders

**DOI:** 10.1186/s40337-016-0099-x

**Published:** 2016-03-15

**Authors:** Phillipa Hay, Stephen Touyz

**Affiliations:** Centre for Health Research, School of Medicine, Western Sydney University, Sydney, Australia; School of Psychology Faculty of Science, University of Sydney, Sydney, Australia

## Editorial

This year’s Editorial began its life in the Antipodean summer in a bay close to where the sounds of bell birds and tui, “the most melodious wild music” first received international attention from records of Cook’s journeys in the eighteenth century [1]. At that time global journeys were dangerous and rare and their scientific discoveries took years to reach a publisher. Now we live in a world community with rapid communications that means that (on a good day) one can communicate to anywhere with an internet connection from the shores Cook took refuge in in 1770.

Eating Disorders, like communications, are now global. By its inherent qualities of open access the Journal of Eating Disorders is in a position to disseminate and reach every corner of the world, and the people within them working in the field of eating disorders who have access to the internet. Alongside this there is an imperative that we understand eating disorders and how to manage them in ways that are helpful beyond our own perspective. In 2015 the *Journal of Eating Disorders* published its first special series on “The Current Status of Eating Disorders: General and Special Population studies”. Many of the papers that were submitted were from Asia and the accompanying review by Pike and Dunne [2] underscores the rise in eating disorders across Asia in particular. Another article in the special series points to the increasing prevalence of eating disorders across all age groups, both sexes and throughout all socio-economic groups in Australia [3]. Furthermore throughout 2015 the Journal’s website conveyed an invitation from the World Health Organization Global Clinical Practice network for readers to contribute their expertise in Eating and Feeding Disorders to the current revisions to the ICD-11 [4]. Whilst similar to the DSM-5 [5] there will be differences in diagnostic criteria reflecting global cultural and clinical diversity. In 2016 researchers and clinicians working across the world thus have the opportunity to have input into the ICD-11 and its final criteria scheduled to be published in 2017.

Along with globalisation and revisions to diagnostic criteria there are increasing challenges to our conceptualisation of “What is an eating disorder”. Pike and Dunne’s [2] review points to the diversity of expression of an eating disorder within population samples in Asia. For example, they report a study which found no body image disorder and/or no fat phobia in a large minority of Japanese women with anorexia nervosa. In their review they also point to the factors of industrialization and urbanization as being as great if not more significant than “Westernization” cultural values in understanding the rise of eating disorders in Asia and other parts of the globe.

Taken together this underscores the degree to which eating disorders classification is in a state of flux and subject to diverse socio and cultural influences. With the Research Domain Criteria (RDoC) [6] movement leading the way we may come closer to an understanding of the neuroscience of appetite, satiety and its relationship to eating and weight facilitating a new understanding of disorders of feeding and eating. We anticipate increasing science in this area written and published in the Journal. It is also important to distil the science and disseminate it to clinicians and the wider community and in this regard the systematic and scientific review is imperative. The Pike and Dunne, and other reviews published in the journal reflect this and for the first time this year we gave a prize both for a primary research paper and for a review paper. We congratulate again our prize winners, for 2015: Dr Loa Clausen [7] and Dr Carmel Harrison [8].

Finally, reflecting the growing globalisation of science, the journal encourages and endeavours to support papers from non-English speaking countries. We recognise however that it is imperative to better develop the processes that allow that to occur, and to have efficient processes in place. Whilst author services such as Edanz [9] are important more needs to be done to guide and support authors.

As part of the 2015 editorial board refreshment we are pleased to welcome Dr Kathleen Pike in a new role for International Affairs and Liaison onto the Advisory Board. We also welcome Dr Janet Treasure and Dr Beth Shelton to our Advisory Board and two new Associate Editors, Dr Ross Crosby and Dr Lois Surgenor. We thank our Managing Editor Mr Jeremy Freeman, Sara Ho at BioMedCentral, and all our Board members, authors and readers. We are delighted to be currently receiving the first submissions of our special issue on Severe and Enduring Anorexia Nervosa. We look forward to continued success in the Journal bringing high quality primary research papers and reviews to researchers and clinicians around the world.

## References

[1] Banks W (1986). Ship’s Naturalist 1770 Quoted in The Story of the Marlborough Sounds National Park Potton C.

[2] Pike KM, Dunne PE (2015). The rise of eating disorders in Asia: a review. J Eat Disord.

[3] Hay P, Girosi F, Mond J (2015). Prevalence and sociodemographic correlates of DSM-5 eating disorders in the Australian population. J Eat Disord.

[4] Al-Adawi S, Bax B, Bryant-Waugh R, Claudino AM, Hay P, Monteleone P, Norring C, Pike KM, Pilon DJ, Herscovici CR, Reed GM (2013). Revision of ICD–status update on feeding and eating disorders. Advances in Eating Disorders.

[5] American Psychiatric Association. Diagnostic and statistical manual of mental disorders (DSM-5®). American Psychiatric Association; 2013 May 22.

[6] Cuthbert BN, Insel TR (2013). Toward the future of psychiatric diagnosis: the seven pillars of RDoC. BMC Med.

[7] Clausen L, Jones A (2014). A systematic review of the frequency, duration, type and effect of involuntary treatment for people with anorexia nervosa, and an analysis of patient characteristics. J Eat Disord.

[8] Harrison C, Mond J, Bentley C, Gratwick-Sarll K, Rieger E, Rodgers B (2014). Loss of control eating with and without the undue influence of weight or shape on self-evaluation: evidence from an adolescent population. J Eating Disords.

[9] Yau L & da Costa T. Raising the acceptance rate of article submissions from ESL authors: A white paper. http://media.biomedcentral.com/content/editorial/WhitePaper_Raising_article_acceptance_ESLauthors.pdf. Downloaded Feb 13 2016.

